# Protective Effect of Neuropeptide Substance P on Bone Marrow Mesenchymal Stem Cells against Apoptosis Induced by Serum Deprivation

**DOI:** 10.1155/2015/270328

**Published:** 2015-05-28

**Authors:** Su Fu, Dan Jin, Song Liu, Lei Wang, Zhao Wang, Gang Mei, Zhen-Lv Zou, Jian-Qun Wu, Zi-Yi Xu

**Affiliations:** ^1^Department of Orthopedics and Traumatology, Nanfang Hospital, Southern Medical University 1838, North Guangzhou Avenue, Guangzhou, Guangdong 510515, China; ^2^School of Engineering and Materials Science, Queen Mary University of London, London, UK; ^3^Department of Orthopaedics, Xiangyang Central Hospital, Xiangyang, Hubei 441021, China

## Abstract

Substance P (SP) contributes to bone formation by stimulating the proliferation and differentiation of bone marrow stromal cells (BMSCs); however, the possible involved effect of SP on apoptosis induced by serum deprivation (SD) in BMSCs is unclear. To explore the potential protective effect of SP and its mechanism, we investigated the relationships among SP, apoptosis induced by SD, and Wnt signaling in BMSCs. SP exhibited a protective effect, as indicated by a reduction in the apoptotic rate, nuclear condensation, caspase-3 and caspase-9 activation, and the ratio of Bax/Bcl-2 that was observed after 24 h of SD. This protective effect was blocked by the inhibition of Wnt signaling or antagonism of the NK-1 receptor. Moreover, SP promoted the mRNA and protein expression of Wnt signaling molecules such as *β*-catenin, p-GSK-3*β*, c-myc, and cyclin D1 in addition to the nuclear translocation of *β*-catenin, indicating that active Wnt signaling is involved in SP inhibition of apoptosis. Our results revealed that mediated by the NK-1 receptor, SP exerts an inhibitory effect on serum deprivation induced apoptosis in BMSCs that is related to the activation of canonical Wnt signaling.

## 1. Introduction

Neuropeptide substance P (SP) is an 11-amino-acid neuropeptide secreted by skeletal sensory neurons [1], which has been demonstrated as the local regulating protein during fracture healing. SP-releasing nerve fibers increase during bone development and repair [2], suggesting the direct involvement of SP in the local regulation of bone formation [3]. Also, a higher degree of osteogenesis was observed when a sensory nerve was implanted in constructing engineered neurotized bone tissue [4, 5], while the phenomenon that increased early expression of neuropeptide receptors as SP after vascular bundles implanting was noticed [4], indicating the possible promoting role of SP acted on BMSCs. In summary, studies both in vivo and in vitro have indicated that SP promotes bone formation by stimulating the proliferation, differentiation, and mineralization of BMSCs [3, 6–8]. The positive function of SP action on BMSCs is based on the balance of proliferation and apoptosis that results in an appropriate number of cells. Studies have shown that SP exerts an inhibitory effect on the apoptotic pathway in human colon cells [9] and radiation-damaged BMSCs [10]. However, the antiapoptotic effect of SP on serum-deprived BMSCs has not been investigated. Thus, in this study, we examined the antiapoptotic effects of SP in serum-deprived rat BMSCs.

The underling mechanism for the potential action of SP on BMSCs is currently under investigation. Multiple pathways, including the RANKL [6] and MAPK pathways [10], are activated by SP to promote the proliferation, differentiation, and apoptosis of BMSCs. Canonical Wnt signaling has been shown to play an important role in bone metabolism [11–14]. The activation of the Wnt pathway can be briefly summarized as requiring the cytoplasmic accumulation and nuclear localization of *β*-catenin prior to the subsequent transcription of Wnt-related genes, including c-myc [15]. Our previous study revealed that the proliferation-promoting effect of SP involves the canonical Wnt pathway [16]. Considering that Wnt signaling acts as the key regulator of diverse processes including apoptosis [17, 18], it is likely that the antiapoptotic effect of SP may be associated with Wnt pathway. Thus, we evaluated whether the antiapoptotic effect of SP is mediated by canonical Wnt signaling.

This study primarily evaluated the hypothesis that SP prevents apoptosis in serum-starved BMSCs via canonical Wnt signaling. The obtained results may aid in understanding the neuroprotective function and mechanism of SP that may be crucial to the process of fracture repair.

## 2. Materials and Methods

### 2.1. Materials

Substance P and NK-1 antagonist were purchased by Sigma (USA); antibodies for *β*-catenin, p-GSK-3*β*, c-myc, and cleaved caspase-3 were obtained from Santa Cruz Biotechnology (Santa Cruz, CA). Cell growth medium was composed of L-DMEM (90%, Gibco, USA) and fetal bovine serum (FBS, 10% v/v, Gibco, USA). The Annexin V-FITC apoptosis detection kit was obtained from KeyGEN Biotech Co., Ltd. (Guangzhou, China).

### 2.2. Preparation of BMSCs

BMSCs from 6-week-old Sprague Dawley rats were harvested using well-characterized techniques [19]. Briefly, after death, both the femur and tibia of the rats were isolated and then aseptically excised with the attached soft tissues and epiphysis removed. Bone marrow was repeatedly flushed out with cell growth medium using a hypodermic needle. After centrifugation at 1000 rpm for 5 min, marrow cells were again flushed into suspension and then plated in 25 cm^2^ tissue culture flasks at a concentration of 1 × 10^6^ cells/mL. The BMSCs were analyzed for the expression of FL1-Height, CD90, IgG1, CD34, CD44, CD45, IgG2a, CD11b/C, HAMSTER, and CD29 via flow cytometry. This method can be demonstrated as excluding the possibility of hematopoietic stem/progenitor cells, fibroblasts, blood cells by detecting CD45, CD34, and including BMSCs to detect the purity by detecting CD44, CD29 [20, 21]. Approximately 5.0 × 104 purified BMSCs were stained with 20 mL phycoerythrin- (PE-) conjugated anti-FL1-Height, CD90, IgG1, CD34, CD44, CD45, IgG2a, CD11b/C, HAMSTER, and CD295 for 30 min at 4°C.

### 2.3. Groups

The harvested BMSCs were transferred to 6-well plates or 96-well plates for subsequent experiments. To evaluate the antiapoptotic effect, the cultures were divided into eight groups that included a control group and SP (10^−8^ mol/L, 10^−10^ mol/L, and 10^−12^ mol/L) groups, each of which was serum deprived for 12 h or 24 h. To clarify the mechanism of this effect, cells were divided into Group A, which was incubated with SP (10^−10^ mol/L); Group B, which was incubated with a combination of SP and an NK-1 antagonist (10^−7^ mol/L) [22]; Group C, which was incubated with a combination of SP and 0.2 *μ*g/mL DKK1 [23]; and Group D, which represented the control group, to which an identical volume of PBS was added. Additional groups, such as cells treated for 15, 30, 60, or 120 min with SP (10^−10^ mol/L) alone or in combination with the SP receptor antagonist spantide (10^−8^ mol/L), DKK1 (0.2 *μ*g/mL), or LiCl (0.02 mol/L) [24], were analyzed to improve the integrity of the study.

### 2.4. Methylthiazolyldiphenyl-Tetrazolium Bromide (MTT) Assay

To explore and quantify the effect of various concentrations and treatment durations of SP on cell viability, an MTT assay was employed. Prepared BMSCs were seeded in 96-well culture plates. SP treatment was initiated after 24 h using different concentrations of SP (0 mol/L, 10^−8^ mol/L, 10^−10^ mol/L, and 10^−12^ mol/L). Because viable cells can absorb and convert the MTT dye into an insoluble reaction product, the optical density reflects the number of viable cells. MTT stock solution (at a 1 : 10 dilution) was added to each well. After incubation at 37°C for 4 h, acidified isopropanol was added to each well and then thoroughly mixed to dissolve the formazan crystals. The plates were subsequently read using a microplate reader at 495 nm.

### 2.5. Apoptosis Assay

Apoptosis of BMSCs induced by serum starvation was assessed using 4′-6′-diamidino-2-phenylindole (DAPI), Annexin V-FITC, and cleaved caspase-3 staining. DAPI was used to characterize nuclear condensation and fragmentation. Collected cells were incubated in DAPI (1 *μ*g/mL) for 5 min at room temperature, washed 3 times with PBS, and then assessed. Photomicrographs of five different randomly chosen fields per well were taken under a phase contrast microscope after incubation. The ratio of apoptotic cells was calculated by dividing the number of positive cells by the total number of cells. The working solution of Annexin V-FITC was prepared immediately prior to use. Treated cells were washed 3 times with PBS and resuspended in binding buffer. Flow cytometric analysis began immediately following incubation with 100 ng/mL Annexin V-FITC and 10 *μ*L of propidium iodide (PI) in the dark for 10 min and 5 mg/mL for 5 min and then washed 3 times with PBS prior to examination. Cleaved caspase-3 staining was also examined. After fixation with 10% formalin for 5 min and permeabilization with 0.2% Triton X-100 for 5 min, cells were blocked with 1% fish gelatin (Sigma) for 1 h at room temperature and incubated with primary antibody overnight. After incubation with secondary antibody for 1 h at room temperature, DAPI was used to stain the nucleus. Dishes were mounted and analyzed under a fluorescence microscope.

### 2.6. qPCR

The Bcl-2, Bax, caspase-3, caspase-8, caspase-9, *β*-catenin, GSK-3*β*, c-myc, and cyclin D1 genes were evaluated in this assay. Total RNA of obtained BMSCs cells was isolated and extracted using the RNeasy Mini Kit purchased in Qiagen (Valencia, CA), and then the purity and concentration of RNA were spectrophotometrically determined. The cDNA with an approximately 20 *μ*L final volume was subsequently synthesized from 1 *μ*g of RNA using an iScript cDNA Synthesis Kit obtained in Bio-Rad Laboratories (Hercules, CA). Real-time PCR reactions were conducted using the SYBR Green PCR Master Mix (Applied Biosystems, Foster City, CA). The primer sequences listed in Table 1 were used in these experiments. To validate the primer sets used in this study, dissociation curves were determined to confirm single-product formation, and agarose gel analysis was conducted to confirm the product size. The data from real-time PCR experiments were analyzed using the comparative CT method as described in the manual for the ABI Prism 7900 Real-Time PCR System. *β*-actin was used as an endogenous reference, and each sample was normalized to the respective *β*-actin level. All experiments were performed in three replicates.

### 2.7. Western Blot Analysis

The caspase-3, *β*-catenin, p-GSK-3*β*, and c-myc proteins were evaluated using Western blot analysis. Cells were extracted using lysis buffer (Cell Signaling Technology) and dissolved in buffer (50 mM Tris–HCl (pH 6.8), 2% SDS, 10% glycerol, and 100 mM dithiothreitol). After BCA evaluation to ensure equivalent loading of protein samples, proteins were separated using 10% SDS-polyacrylamide gel electrophoresis (SDS-PAGE), transferred to a nitrocellulose membrane, and probed with anti-cleaved caspase-3 antibodies (Abcam, CA), anti-*β*-catenin antibodies, anti-p-GSK-3*β* antibodies, and anti-c-myc antibodies (Santa Cruz Biotechnology, Santa Cruz, CA). ECL reagents (Amersham Biosciences, Piscataway, NJ) were used for detection. Bands were quantified using densitometry of digitized images. Each blot was subsequently stripped and reprobed using anti-*β*-actin antibodies for normalization of the expression.

### 2.8. Immunofluorescence Staining

Grouped cells were seeded and cultured in 96-well plates to be tested with immunofluorescence staining of *β*-catenin. With DMEM moved, prepared BMSCs were firstly fixed in 4% paraformaldehyde in PBS during 15 min at moderate room temperature. After permeabilization in 0.25% Triton X-100 in PBS for 15 min, cells were incubated in 1% BSA in PBST for 30 min to avoid nonspecific binding of the antibody, washed in PBS for three times, and incubated overnight with primary anti-*β*-catenin antibody purchased from Santa Cruz Biotechnology, Inc., diluted 1 : 100 in PBST. After three washes, the cells were incubated for 1 h with FITC-linked secondary antibodies diluted 1 : 100 (USCN). Cells were washed three times in PBS and then DAPI stained to identify nuclei for the detection of *β*-catenin translocation. Slides were examined by fluorescence microscopy, and images were acquired using Image Manager software.

### 2.9. Statistical Analysis

GraphPad Prism (GraphPad Prism 5.01, GraphPad Software, San Diego, CA, USA) was used to perform the statistical analysis. Obtained data was acquired from MTT assays, flow cytometric analysis, qPCR, and Western blot analysis which are presented as the means ± SD and were analyzed using one-way ANOVA followed by the Bonferroni test. All error bars indicate the calculated standard error of the mean (SEM). Significance was set at *P* < 0.05 (indicated by asterisks in figures). In histograms, asterisks above bars indicate significance in comparison to the control group.

## 3. Results

### 3.1. BMSCs Preparation

**Figure 1:**
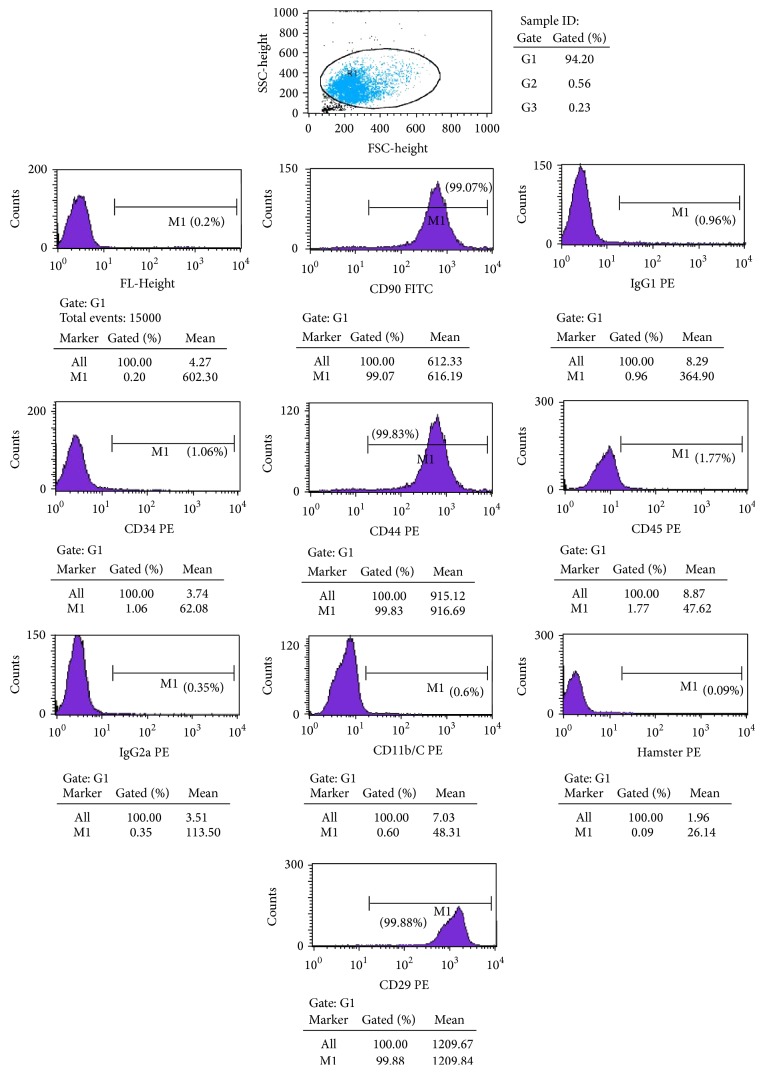
Preparation of BMSCs. The cultured cells were identified with FL1-Height (0.2% ± 1.7%), CD90 (99.7% ± 3.1%), IgG1 (0.96% ± 1.3%), CD34 (1.06% ± 1.3%), CD44 (99.8% ± 4.1%), CD45 (1.7% ± 2.1%), IgG2a (0.35% ± 2.9%), CD11b/C (0.6% ± 1.7%), HAMSTER (0.09% ± 0.7%), and CD29 (99.88% ± 3.1%).

The cultured BMSCs were examined under an optical microscope with a spindle shaped morphology. Following passage of the third generation culture, cells grew faster than the original generation. The results of the flow cytometry showed that the cultured cells were FL1-Height (0.2% ± 1.7%), CD90 (99.7% ± 3.1%), IgG1 (0.96% ± 1.3%), CD34 (1.06% ± 1.3%), CD44 (99.8% ± 4.1%), CD45 (1.7% ± 2.1%), IgG2a (0.35% ± 2.9%), CD11b/C (0.6% ± 1.7%), HAMSTER (0.09% ± 0.7%), and CD29 (99.88% ± 3.1%), which indicated that the cells were BMSCs (Figure 1).

### 3.2. SP Protects BMSCs from SD-Induced Apoptosis

**Figure 2:**
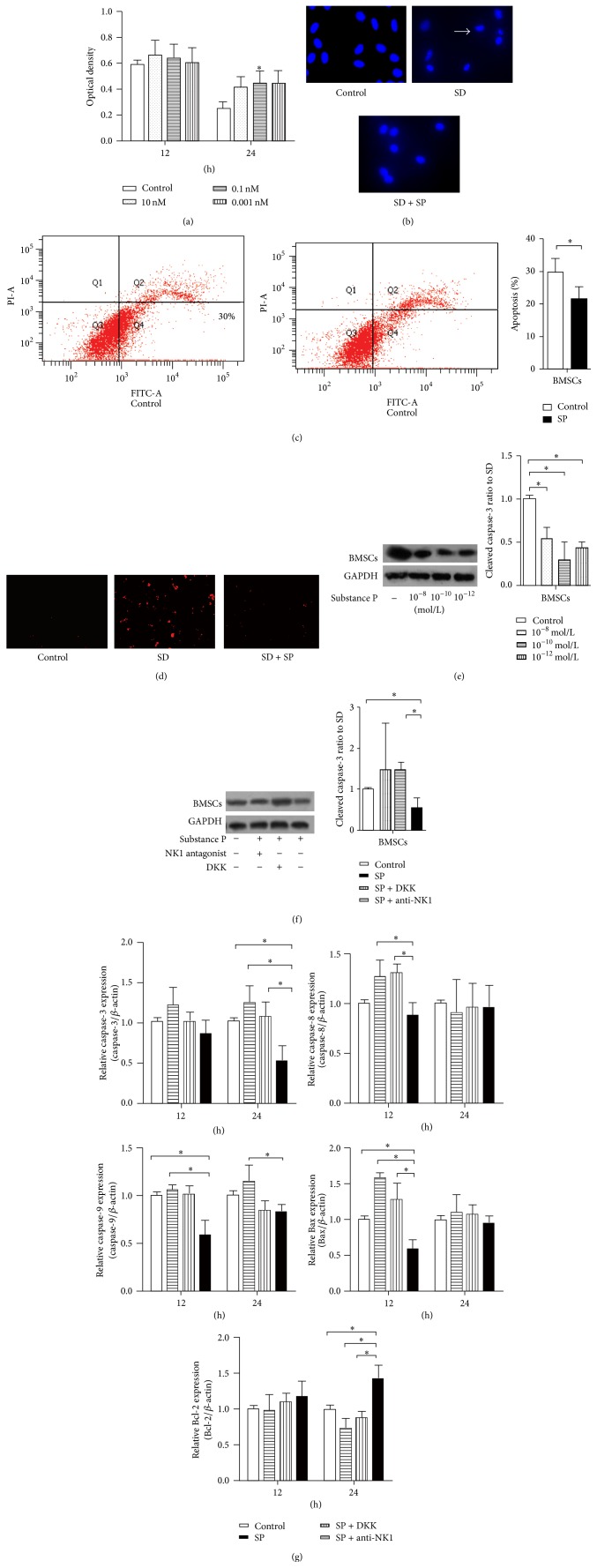
Effect of SP on apoptotic changes in BMSCs. MTT assays and DAPI staining were used to quantify cell viability and to detect nuclear changes. A trend of SP reduction of SD-induced apoptotic cell death was observed from the MTT assays, although SP did not exhibit a comprehensively significant prevention in BMSCs (a). Although the concentrations ranging from 10^−8^ to 10^−12^ mol/L all exhibited a protective trend, only 10^−10^ mol/L SP exhibited a significant difference in comparison to the control group in BMSCs after 24 h of SD. Images of DAPI staining were taken 24 h after treatment and indicate that the nuclear condensation and integrity changes of BMSCs were reduced by SP (b). Arrows mark shrunken nuclei that indicate apoptotic cells. The flow cytometric analysis of Annexin V-FITC showed the protective trend of 10^−10^ mol/L SP function at 24 h SD treatment (c). Multiple images illustrate the presence of cleaved caspase-3 in BMSCs control cells and in cells subjected to 24 h SD or SD plus SP treatment. The red immunostaining of cleaved caspase-3 suggested a decrease in caspase-3-positive cells under SP treatment (d). Caspase-3 activation was quantified using Western blot analysis in the concentration manner (e) or combined with the treatment of DDK or NK-1 antagonist (f); a significant reduction in caspase-3 mRNA is indicated by qPCR (g) after 24 h of SD in BMSCs. Also, the mRNA levels of the apoptotic molecules Bcl-2, Bax, caspase-8, and caspase-9 were analyzed using qPCR in BMSCs (g). Although a clear decrease in Bax expression was not observed, the ratio of Bcl-2 to Bax was increased due to SP promotion of Bcl-2 expression. Caspase-8 expression was essentially identical between the SP group and the control group, and caspase-9 expression was reduced by SP treatment.

To investigate the antiapoptotic effects of SP, cell viability and apoptosis-related cell changes were examined. MTT assays indicated that cell viability was protected by SP in a time- and concentration-dependent manner. After 12 h of SD, a significant difference was not observed between the treated and untreated cells for BMSCs; the protective trend was clearly observed in BMSCs only after 24 h of SD (Figure 2(a); 0.248 ± 0.0512 in the control group versus 0.446 ± 0.093 in the SP 10^−10^ mol/L group). Based on the results of the MTT assay, the 24 h time point and 10^−10^ mol/L SP concentration were selected for subsequent experiments. SD during 24 h caused the typical increase in apoptosis (31.7 ± 3.1% in BMSCs) and nuclear condensation; some cells were even fragmented. DAPI staining indicated that SP could partially block nuclear changes such as condensation and deformation in comparison to the control group. SD-induced apoptosis following SP treatment was significantly reduced (20.3 ± 5.2% in BMSCs; Figure 2(c)) as indicated by Annexin V-FITC staining, and this evident effect was ascertained as protective. These results suggest that SP can inhibit SD-induced apoptosis in BMSCs.

The effect of SP on apoptotic molecules was also evaluated. SP prevented the activation of caspase-3 after 24 h of SD as assessed by cleaved caspase-3 staining, qPCR, and Western blot analysis (Figure 2). Increased cleaved-caspase-3-positive cells were observed in the SD group, whereas SP inhibited this activation and reduced the percentage of cleaved-caspase-3-positive cells. This finding may result from a substantial reduction in caspase-3 mRNA levels (the relative mRNA expression for the SP group was 0.506 ± 0.126) and protein expression (the relative protein expression of the SP group was 0.506 ± 0.126) by SP. SP at a concentration of 10^−10^ mol/L maximally decreased the level of active caspase-3, as observed for 10^−12^ mol/L SP. A decreasing trend in the mRNA expression of Bax and caspase-9 and an increasing trend in the mRNA expression of Bcl-2 were clearly observed (Figure 2(g)). The expression of the Bax gene was reduced by SP after 12 h of SD in BMSCs; this effect was blocked by treatment with an NK-1 antagonist or DKK1. SP treatment increased Bcl-2 gene expression after 24 h in BMSCs (the relative Bcl-2 expression in the SP group was 1.424 ± 0.192 in BMSCs). In summary, SP treatment elevated the important ratio of Bcl-2 to Bax and reduced caspase-9 synthesis, as clearly indicated by qPCR. Although caspase-8 mRNA expression was not clearly affected by SP, increased caspase-8 mRNA expression was observed in BMSCs after 12 h when treated with an NK-1 antagonist or DKK1.

### 3.3. Roles of the Wnt Pathway in Mediating SP Effects

**Figure 3:**
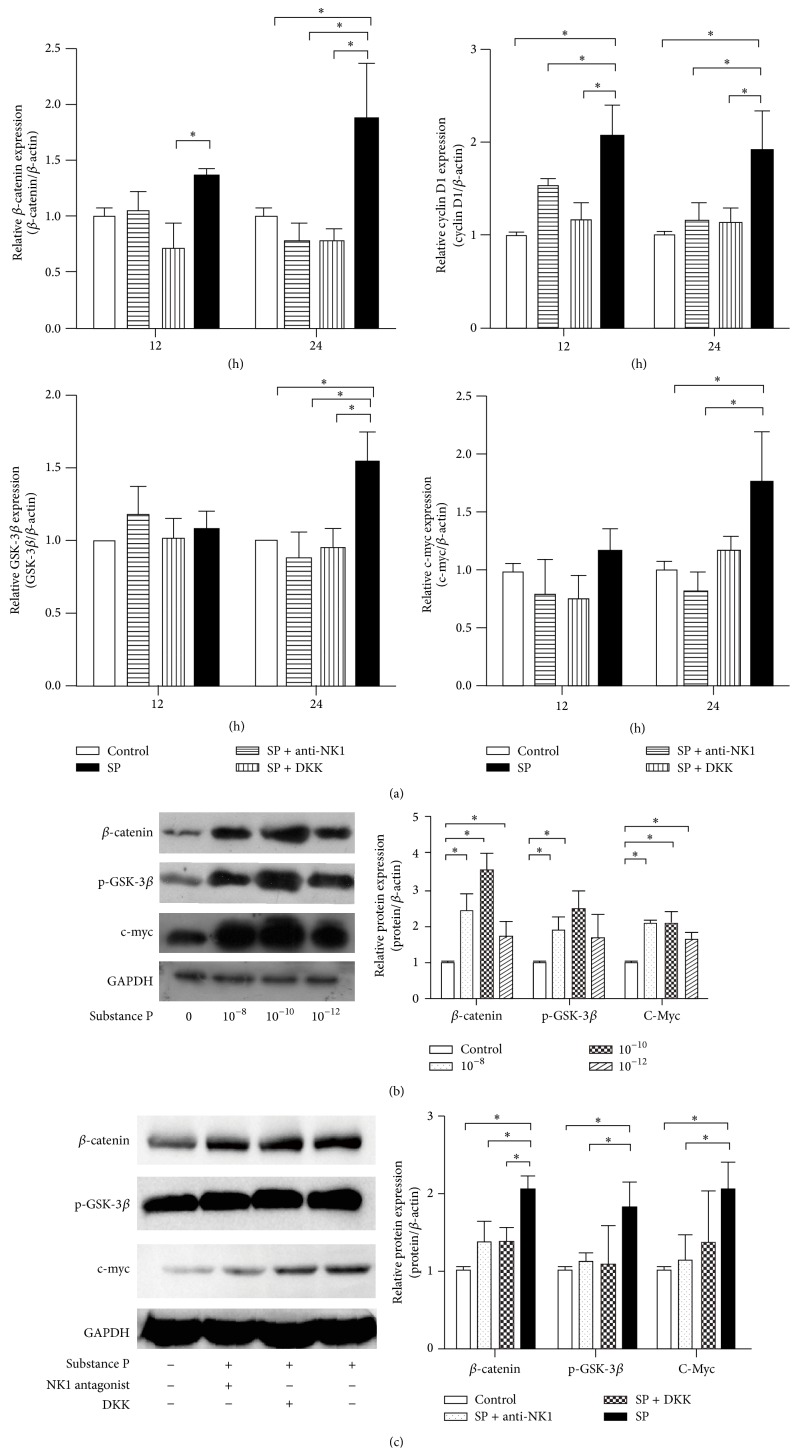
Effect of SP on Wnt-related molecules in BMSCs. The mRNA and protein levels of the Wnt pathway proteins *β*-catenin, GSK-3*β*/p-GSK-3*β*, cyclin D1, and c-myc were evaluated by qPCR and Western blot analysis in BMSCs. A significant activating effect of SP on Wnt signaling was observed after 12 h (a). Various concentrations of SP (10^−8^, 10^−10^, and 10^−12^ mol/L) upregulated the protein levels of Wnt pathway proteins (b). An NK-1 antagonist and the Wnt pathway inhibitor DKK1 both attenuated the activating effect of SP on Wnt signaling, indicating that the Wnt pathway mediates the effect of SP (c).

**Figure 4:**
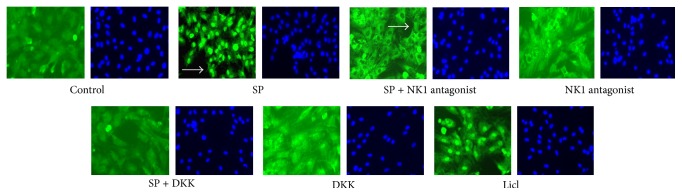
Effect of SP on the nuclear translocation of *β*-catenin in BMSCs. SP exhibited a stable ability to activate *β*-catenin nuclear translocation. In BMSCs, nuclear *β*-catenin was not evident, whereas its nuclear localization was clearly observed following SP treatment, as indicated by FITC-linked *β*-catenin (indicated by the arrow in the SP group). With combined NK-1 antagonist and SP treatment, nuclei appeared relatively dark (indicated by the arrow in the SP + NK-1 antagonist group).

Canonical Wnt signaling proteins as *β*-catenin, p-GSK-3*β*, cyclin D1, and c-myc were served as the critical molecules, which were assessed by using qPCR and Western blot analysis (Figure 3). SP treatment clearly elevated the expression levels of Wnt pathway mRNAs, which were attenuated by an NK-1 antagonist or DKK1 treatment, particularly significant after 24 h SD treatment. Expression of *β*-catenin was strongly induced by SP, but this effect was only significant in BMSCs after 24 h of treatment. The elevated mRNA expression levels of GSK-3*β* and c-myc in SP treatment group were higher than the expression levels in control group after 24 h of SD. All the concentrations of SP all affected the expression of Wnt signaling molecules. The immunofluorescence staining of *β*-catenin transforming to nuclear illustrated the effect of SP on Wnt pathway. It was the relatively low abundance in the nucleus that the cells in control group were filled with. The nuclear became apparent after SP treatment, but this effect was not observed after pretreatment with an NK-1 antagonist or DKK1 (Figure 4).

## 4. Discussion

SP has been demonstrated to be a promoting factor in BMSCs at different levels, such as proliferation, differentiation, and cellular function, and it has been recommended as a drug to treat osteoporosis [7, 9, 16, 25]. However, the effect of SP and its mechanism on BMSCs subjected to SD has not been investigated, although SP reportedly exhibits the ability to suppress apoptosis in various other cell types via different pathways [9, 10, 25, 26]. Thus, this study aimed to examine the anabolic effect of SP on SD-induced rat BMSCs. We demonstrate that the protective effect of SP, which was dependent on SP concentration and treatment time, was mediated by the NK-1 receptor of SP that is involved in decreasing the Bax-to-Bcl-2 ratio and the mRNA expression of caspase-3. Moreover, the induction of mRNA and protein expression of Wnt pathway proteins such as *β*-catenin, c-myc, and GSK-3*β* in addition to the nuclear translocation of *β*-catenin may contribute to this effect. Our observations primarily complement the effect and mechanism of the endogenous SP peptide.

Serum deprivation is commonly used to induce apoptosis to understand typical apoptotic changes [27, 28]. The advantage of this in vitro study is the utilization of a precise apoptotic model of the classical apoptotic changes to explore the action of SP; however, such a model cannot evaluate the in vivo effect on mixed types of cell death. Different from the previous research exploring the effect of reducing radiation induced BMSC apoptosis, the serum deprivation condition simulated the cells lacking nutrition provided as the fracture area. The optimal working concentration of SP is in dispute; SP at 10^−12^ mol/L stimulated alkaline phosphatase synthesis in osteoblast differentiation, whereas a higher concentration (10^−8^ mol/L) stimulated mineralization [6]. SP at 10^−8^ and 10^−10^ mol/L promoted the proliferation of rat BMSCs [3], whereas SP at 10^−7^ mol/L protected BMSCs from irradiation damage [10], and SP treatment at 10^−8^ mol/L to 10^−6^ mol/L increased the size and number of bone colonies [8]. In light of the different previously used doses, we first determined an appropriate SP concentration using MTT assays, which was confirmed using qPCR and Western blot analysis. Based on our results and considering the SP concentration in humans [29–31], the concentration we selected is close to the physiological level and is able to protect cells from apoptosis. The dose-dependent and time-dependent survival effect of SP was demonstrated using multiple methods. MTT assays indicated that the trend was at least maintained after 12 h and 24 h, although the optimal SP concentrations was the only obvious effect (10^−10^ mol/L SP demonstrated the strongest effect after 24 h). Although the trend was not significant, particularly for the 12 h treatment (which is likely due to mixed types of cell death during the onset of SD), a protective trend was observed in addition to the attenuation of nuclear changes induced by SP.

The antiapoptotic effect was finally confirmed by flow cytometry and the determination of the mRNA expression of apoptotic molecules. Consistent with the protective mechanism of SP against radiation damage of BMSCs [10], clearly altered mRNA levels of Bcl-2, caspase-3, and caspase-9 induced by SP were also observed in our study. A decreased ratio of Bcl-2 to Bax can induce apoptosis by enhancing mitochondrial apoptotic signaling upstream of caspase-9, which then activates caspase-3 that results in apoptotic changes [32]. Thus, SP acted on BMSCs to attenuate apoptosis primarily by decreasing mitochondrial apoptotic signaling. Although the trend of this effect was similar to the previous research results [10], the changes in apoptotic molecules were not completely identical due to their different detecting methods but primarily due to the experimental interventions. In serum-deprived BMSCs, the decreased Bax mRNA level after 12 h and increased Bcl-2 mRNA level after 24 h were evident, thereby elevating the Bcl-2-to-Bax ratio to attenuate apoptosis. Overexpression of Bax can regulate the mitochondrial permeability transition to promote apoptosis, and, conversely, overexpression of Bcl-2 can prevent apoptotic cell death [20, 33]. Thus, the ratio of Bax to Bcl-2 determines cell fate in response to apoptotic stimuli [21]. As expected, the levels of Bax mRNA expression, cleaved caspase-3, and the mRNA expression other key apoptosis molecules were significantly reduced in response to SP treatment, whereas Bcl-2 mRNA expression increased. Although the Bcl-2 mRNA level did not differ between the control and SP groups, the ratio of Bax to Bcl-2 was lower in the SP group. Therefore, these results indicate that SP reduces apoptosis in BMSCs by promoting Bcl-2 synthesis in contrast to Bax. However, Bax and caspase-8 mRNA expression were not significantly attenuated, which may be due to the activation of intrinsic antiapoptotic mechanisms. Additionally, the mRNA detected by qPCR is not always an indicator of the existing protein level. Thus, the trend observed for Bax and caspase-8 mRNA expression, although not significant, may reflect the action of SP.

The canonical Wnt pathway has been activated by SP in order to promote the proliferation and differentiation of BMSCs [16, 34, 35]. This current study illustrated the active canonical Wnt pathway could be induced by SP in the serum-deprived BMSCs. The p-GSK-3*β* proteins which acted as the regulating factor of *β*-catenin were elevated by SP treatment in the three concentrations, which was identically referred to the proteins of *β*-catenin and c-myc. The observation that the mRNA expression of apoptotic molecules increased in the presence of DKK1 and SP compared with SP alone also indicates the role of Wnt signaling in the SP-induced effect. These evidences revealed the relationship of Wnt signal involved in the SP function but further experiments both in vitro and in vivo will elucidate the effect of SP.

It can be hypothesized that the function of SP in bone is to reduce apoptosis. The modulation of the Wnt pathway in BMSCs by SP confirms that SP plays a role in bone physiology and promotes survival via Wnt signaling. Activation of the Wnt pathway in mice results in decreased osteoblast apoptosis [13]. Our study suggests that SP exhibited an antiapoptotic effect on serum deprivation-induced apoptosis in BMSCs. Therefore, SP treatment of osteoblasts may also exhibit similar antiapoptotic effects. Considering the critical role of apoptosis during bone formation and reconstruction and the critical effect of Wnt pathway activity during fracture healing, it is likely that the antiapoptotic effect of SP via Wnt signaling is involved in the overall action of this peptide on bone. SP, as observed for CGRP released by nerve fibers [24], exhibited a protective function and demonstrates the neuroprotective effect of neuropeptides. In combination with the protective effect of neuropeptides such as CGRP [36] and SP, the role of neuropeptides can be more comprehensively understood.

In summary, this study demonstrated that SP could activate Wnt signaling by promoting the expression of genes and proteins such as *β*-catenin and c-myc and by promoting the nuclear translocation of *β*-catenin, thereby improving the survival of BMSCs. The detailed gene and protein events that may be crucial for the effect of SP have been partially characterized.

## Figures and Tables

**Table 1:** Primers used for qPCR.

Target gene (rat)	Primer sequence	Predicted length (bp)
*β*-catenin	F: CTCCCCTGACAGAGTTGCT R: ATGTCCAGTCCGAGATCAGC	187

CCND1	F: GCGTACCCTGACACCAATCT R: CTCTTCGCACTTCTGCTCCT	180

c-Myc	F: GCTCCTCGCGTTATTTGAAG R: TTCTCTTCCTCGTCGCAGAT	152

GSK-3*β*	F: TCCGATTGCGGTATTTCTT R: TCACAGGGAGTGTCTGCTT	138

Bcl-2	F: AGTACCTGAACCGGCATCTG R: CAGGTATGCACCCAGAGTGA	173

Bax	F: CGAGCTGATCAGAACCATCA R: CTCAGCCCATCTTCTTCCAG	191

Caspase-3	F: GGACCTGTGGACCTGAAAAA R: GCATGCCATATCATCGTCAG	159

Caspase-8	F: CTGGGAAGGATCGACGATTA R: TGGTCACCTCATCCAAAAC	100

Caspase-9	F: CTCAGGCCAGAGGTTCTCAC R: GGGCAGAAGTTCACGTTGTT	173
